# Longitudinal Intention Tremor Trajectories in Essential Tremor: A Comment

**DOI:** 10.5334/tohm.1193

**Published:** 2026-06-05

**Authors:** Muhammad Saqib Khan, Syed Huzaifa Khan

**Affiliations:** 1 Khyber Medical University, Peshawar, Pakistan; 2 Nowshera Medical College, Nowshera, Pakistan

**Keywords:** Intention Tremor, Essential Tremor, Longitudinal Study

## Abstract

We read with interest the recent prospective study by Louis et al. examining longitudinal changes in intention tremor severity in essential tremor. The study provides valuable data addressing an important and previously understudied question regarding whether intention tremor worsens over time. While the findings demonstrate a significant group-level increase in intention tremor severity, we urge caution in extending these clinical observations to conclusions about progressive cerebellar degeneration. In the absence of direct structural, imaging, or pathological evidence, longitudinal progression of a cerebellar-related clinical sign does not necessarily confirm underlying neurodegenerative changes. Furthermore, the substantial heterogeneity observed in tremor trajectories, with a considerable proportion of patients showing stable or improved intention tremor, suggests that progression is not uniform and may reflect phenotypic variability within essential tremor rather than a universal degenerative process. Future studies incorporating direct measures of cerebellar structure and function, along with longer follow-up and phenotypic stratification, will be important to clarify the relationship between intention tremor progression and underlying cerebellar pathology.

Dear Editor,

We read with interest the recent article by Louis et al. examining the longitudinal trajectory of intention tremor in patients with essential tremor [1]. The authors should be commended for conducting a prospective study addressing an important and understudied question, namely whether intention tremor severity changes over time in essential tremor. The findings contribute valuable longitudinal data to the literature. We would, however, like to offer several comments regarding the interpretation of these findings, particularly with respect to the conclusions drawn about progressive cerebellar degeneration.

The authors suggest that worsening intention tremor over time supports a model of progressive cerebellar degeneration in essential tremor. While the authors appropriately acknowledge several of these limitations in their Discussion, the interpretive extension from longitudinal change in a clinical sign to progressive cerebellar degeneration warrants additional caution. In the absence of direct measures of cerebellar structure or function, such as neuroimaging, physiological markers, or pathological data, the observed changes more directly demonstrate progression of a cerebellar-related clinical sign rather than progressive cerebellar damage itself. As prior work has shown mixed and inconsistent evidence regarding structural cerebellar changes in essential tremor, caution is warranted when extending clinical progression of intention tremor to inferences about underlying neurodegeneration [2,3].

An additional consideration is the marked heterogeneity in longitudinal intention tremor trajectories observed in the cohort. Although a group-level time effect was present, nearly half of the patients demonstrated stable or improved intention tremor severity over follow-up. This variability suggests that progression of intention tremor is not uniform across individuals with essential tremor and may not represent an intrinsic or defining feature of the disorder in all cases. Rather, these findings may be more consistent with phenotypic heterogeneity within essential tremor, with differing trajectories of cerebellar-related signs across patients, an interpretation that has been increasingly emphasized in contemporary models of essential tremor [4].

In summary, this prospective study provides valuable longitudinal data on intention tremor in essential tremor and represents an important contribution to the field. Our comments are intended to highlight the need for caution in extending clinical progression of intention tremor to inferences about progressive cerebellar degeneration, particularly in light of the observed heterogeneity across patients. Future studies incorporating direct measures of cerebellar structure and function, alongside longer follow-up and phenotypic stratification, will be essential to further clarify the relationship between intention tremor progression and underlying cerebellar pathology in essential tremor.
